# Different Potential of Extracellular Vesicles to Support Thrombin Generation: Contributions of Phosphatidylserine, Tissue Factor, and Cellular Origin

**DOI:** 10.1038/s41598-017-03262-2

**Published:** 2017-07-26

**Authors:** Carla Tripisciano, René Weiss, Tanja Eichhorn, Andreas Spittler, Thomas Heuser, Michael Bernhard Fischer, Viktoria Weber

**Affiliations:** 1 Christian Doppler Laboratory for Innovative Therapy Approaches in Sepsis, Danube University Krems, Dr.-Karl-Dorrek-Strasse 30, 3500 Krems, Austria; 2 Core Facility Flow Cytometry & Surgical Research Laboratories, Medical University of Vienna, Lazarettgasse 14, 1090 Vienna, Austria; 3 Electron Microscopy Facility, Vienna Biocenter Core Facilities, Dr.-Bohr-Gasse 3, 1030 Vienna, Austria; 4 Center for Biomedical Technology, Department for Health Sciences and Biomedicine, Danube University Krems, Dr.-Karl-Dorrek-Strasse 30, 3500 Krems, Austria

## Abstract

Cells release diverse types of vesicles constitutively or in response to proliferation, injury, inflammation, or stress. Extracellular vesicles (EVs) are crucial in intercellular communication, and there is emerging evidence for their roles in inflammation, cancer, and thrombosis. We investigated the thrombogenicity of platelet-derived EVs, which constitute the majority of circulating EVs in human blood, and assessed the contributions of phosphatidylserine and tissue factor exposure on thrombin generation. Addition of platelet EVs to vesicle-free human plasma induced thrombin generation in a dose-dependent manner, which was efficiently inhibited by annexin V, but not by anti-tissue factor antibodies, indicating that it was primarily due to the exposure of phosphatidylserine on platelet EVs. Platelet EVs exhibited higher thrombogenicity than EVs from unstimulated monocytic THP-1 cells, but blockade of contact activation significantly reduced thrombin generation by platelet EVs. Stimulation of monocytic cells with lipopolysaccharide enhanced their thrombogenicity both in the presence and in the absence of contact activation, and thrombin generation was efficiently blocked by anti-tissue factor antibodies. Our study provides evidence that irrespective of their cellular origin, EVs support the propagation of coagulation via the exposure of phosphatidylserine, while the expression of functional tissue factor on EVs appears to be limited to pathological conditions.

## Introduction

Extracellular vesicles are released under physiological and pathological conditions and have been detected in all body fluids, including peripheral blood. Based on their biogenesis, diameter, and membrane markers, they are commonly classified into endosome-derived exosomes (30–150 nm), plasma membrane-derived microvesicles (100–1000 nm; also referred to as microparticles or ectosomes), and apoptotic bodies (1000–3000 nm). Since an objective discrimination of these vesicle types is currently hampered by a lack of reliable separation and characterization methods, and their nomenclature is still being refined by the research community^1, 2^, we will use the general term extracellular vesicles (EVs) in the context of this study.

Elevated levels of platelet-derived EVs, which represent the most abundant EV population in peripheral blood^3, 4^, have been described in pathologies associated with an increased risk of thromboembolic events, including cancer, atherosclerosis, sepsis, and pre-eclampsia^5–7^. EVs support coagulation via the exposure of phosphatidylserine, providing a catalytic surface to facilitate the formation of the tenase (factors VIIIa, IXa, and X) and prothrombinase (factors Va, Xa, and II) complexes of the coagulation cascade. Phosphatidylserine may additionally contribute to the transformation of tissue factor from its inactive, encrypted form into a biologically active state^8–10^. Tissue factor (factor III, CD142) is a 263-amino acid polypeptide consisting of an extracellular N-terminal domain, a transmembrane domain, and a cytoplasmic C-terminus. It has a calculated molecular weight of 24.4 kDa, but migrates with an apparent molecular weight of 45–55 kDa in SDS-PAGE due to N-glycosylation at Asn residues 11, 124, and 137. By binding to FVII/FVIIa, tissue factor forms the TF/FVIIa complex, the main physiological initiator of the coagulation cascade. Tissue factor is constitutively expressed by the subendothelium, forming a hemostatic envelope separated by the endothelium from circulating FVII/VIIa to prevent inappropriate coagulation. To ensure a rapid response to injury, however, there might be a low basal activation or idling of the coagulation system, which has been proposed to be induced by low levels of blood-borne tissue factor^11^.

Monocytes and endothelial cells do express tissue factor under certain pathological conditions or upon stimulation with endotoxin or cytokines^12^, but the presence of biologically active tissue factor on neutrophils, platelets, or EVs remains controversial^13–15^. There have been reports on the synthesis of functional tissue factor by non-activated platelets with an increase after platelet activation^16, 17^, on the storage of tissue factor within platelet α-granules^18^, or on its transfer from monocytes to platelets^19^, while other studies found no indication for the presence of functional tissue factor on platelets^20–23^. The use of different antibody clones for tissue factor detection may at least partly account for these divergent results^24, 25^.

Here, we isolated EV fractions from platelet concentrates from healthy donors to investigate their phosphatidylserine and tissue factor-dependent pro-coagulant activity, and we compared the thrombogenicity of platelet-derived EVs to EVs from non-stimulated and lipopolysaccharide-stimulated monocytic cells.

## Results

### Characterization of Platelet Concentrates

Medical grade platelet concentrates obtained from healthy donors in a blood bank setting were used as starting material to isolate EV fractions. On average, they contained 1.9 × 10^6^ platelets/µl, 8 × 10^4^ erythrocytes/µl, and 4 × 10^1^ leukocytes/µl (n = 12). Platelet activation was monitored by flow cytometric analysis of P-selectin (CD62P) surface expression, with an average of 12 ± 2% CD62P^+^ platelets in the platelet concentrate (Supplementary Figure S4a).

EVs were characterized by flow cytometry as described in the Methods section, and staining with annexin V or lactadherin was used to identify phosphatidylserine exposing EVs. Platelet concentrates contained an average of 2 × 10^5^ EVs/µl. About 10% of these EVs (21,660 ± 9473 EVs/µl) were annexinV^+^, and 88.4% of all annexinV^+^ EVs were platelet-derived (CD41^+^annexinV^+^), while 2.1% were erythrocyte-derived (CD235a^+^annexinV^+^), 0.9% were leukocyte-derived (CD45^+^annexinV^+^), 0.6% were derived from monocytes (CD14^+^annexinV^+^), and 8.2% were of unspecified cellular origin (annexin V single positive).

### Isolation of Extracellular Vesicles by Sequential Centrifugation

**Figure 1:**
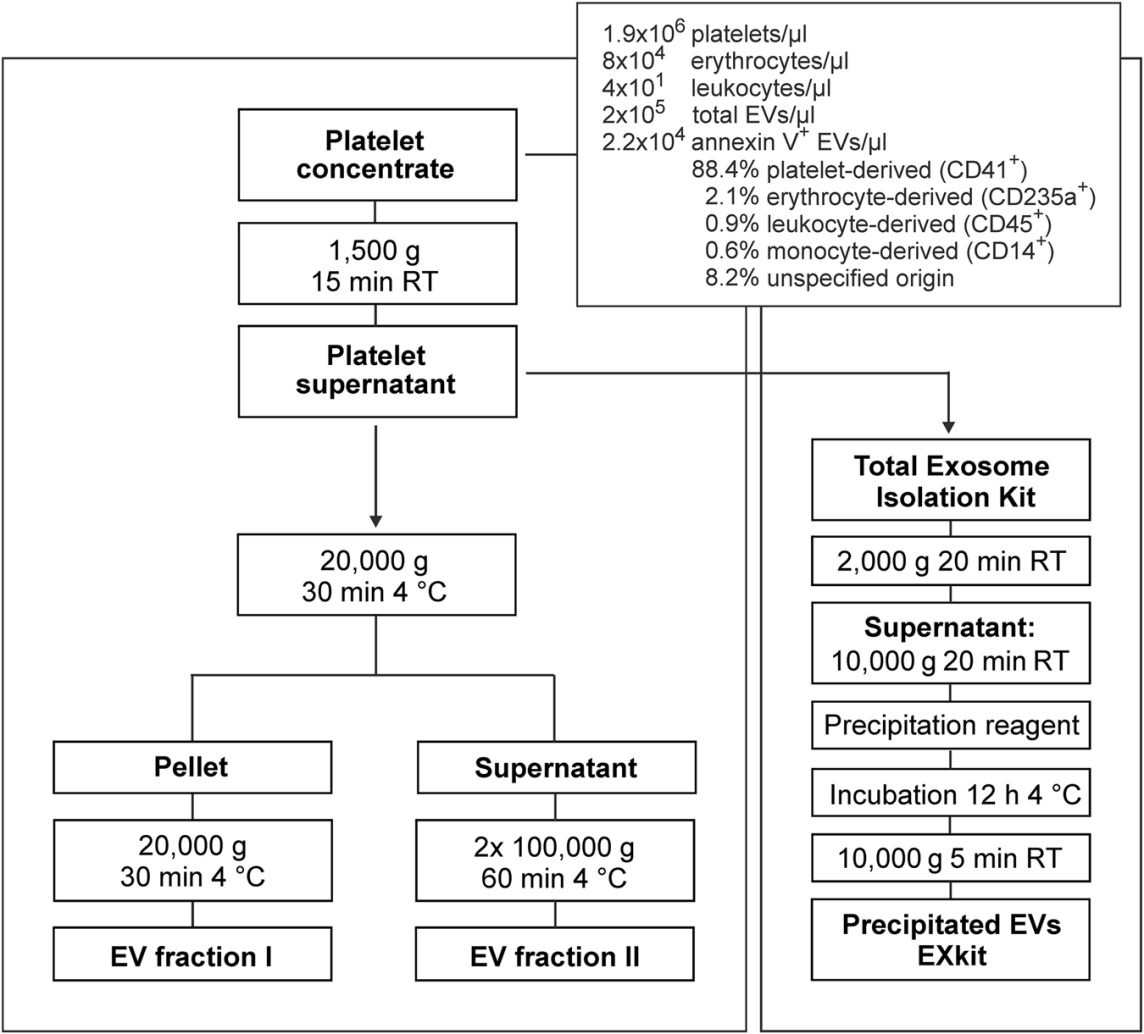
Isolation of extracellular vesicles (EVs). EV fractions were isolated from fresh medical grade platelet concentrates from healthy donors. After removal of platelets by centrifugation at 1,500 g, EVs were obtained by sequential centrifugation of the supernatant at 20,000 g and 100,000 g, respectively, yielding EV fraction I and EV fraction II. For comparison, polymer precipitation using a commercial exosome isolation kit was applied, yielding the EXkit fraction. RT, room temperature.

**Figure 2:**
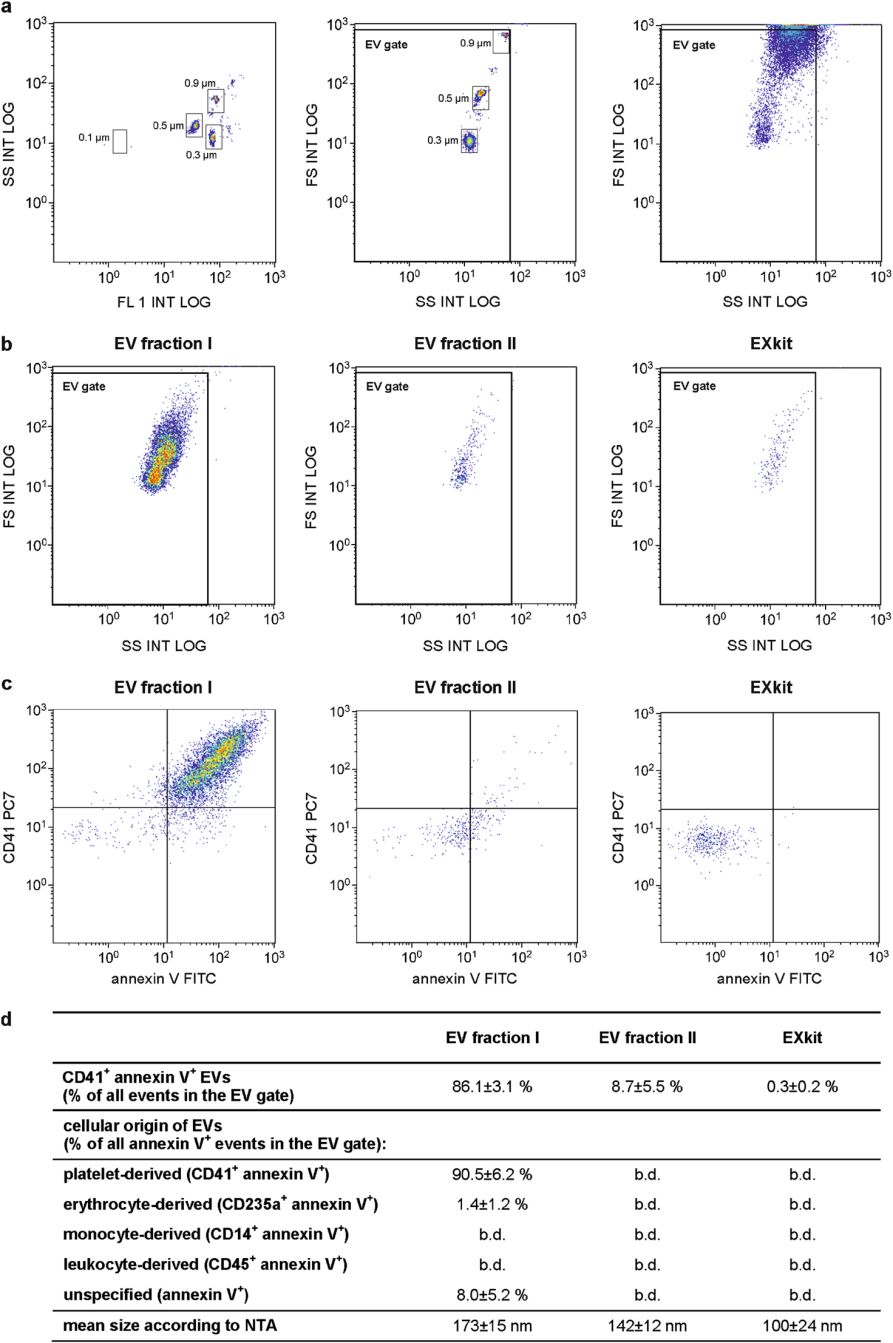
Flow cytometric characterization of isolated EV fractions. EVs were isolated from platelet concentrates as shown in Fig. 1. (**a**) Flow cytometric characterization was performed after calibration with fluorescent beads (0.1, 0.3, 0.5, and 0.9 µm; left panel), and the EV gate was set above the 0.9 µm bead cloud as described in the Methods section (middle panel). A forward scatter *vs*. side scatter (FS *vs*. SS) dot plot for platelet concentrate is shown as example (right panel). (**b**) EV fractions obtained by differential centrifugation of the platelet supernatant at 20,000 g and 100,000 g (EV fraction I and II) or by polymer precipitation (EXkit) are shown in FS *vs*. SS dot plots. (**c**) Characterization of EVs using CD41 as platelet marker and annexin V as marker for phosphatidylserine exposing EVs. Forward scatter *vs*. side scatter plots as well as CD41 *vs*. annexin V scatter plots are shown for all EV fractions. (**d**) Characterization of the EV fractions, specification of the cellular origin of EVs in fraction I, and average vesicle size in EV fractions according to nanoparticle tracking analysis (NTA). The following markers were used to specify the cellular origin of EVs: CD41 (platelet), CD235a (erythrocyte), CD14 (monocyte), and CD45 (leukocyte). The flow cytometric detection limit of 250 nm prohibited a reliable differentiation of EVs in fraction II and in the EXkit fraction with respect to their cellular origin. Data represent the mean of 6 independent isolation experiments ± standard deviation. b.d., below the detection limit.

EV fractions were isolated from platelet concentrates by sequential centrifugation as shown in Fig. 1. For comparison, polymer precipitation using a commercial exosome isolation kit was applied. The platelet supernatant obtained by centrifugation of the platelet concentrate at 1,500 g contained 19.7 ± 9.2% CD41^+^annexinV^+^ events in the EV gate. Sequential centrifugation at 20,000 g and 100,000 g yielded EV fraction I containing 86.1 ± 3.1% CD41^+^annexinV^+^ events in the EV gate, and EV fraction II containing 8.7 ± 5.5% CD41^+^annexinV^+^ events. Vesicles enriched from the same starting material by polymer precipitation with the exosome isolation kit (EXkit fractions) contained only 0.3 ± 0.2% CD41^+^annexinV^+^ events in the EV gate (Fig. 2a,b).

Flow cytometry confirmed that platelets were the main source of EVs in fraction I, which contained only marginal amounts of erythrocyte-derived (CD235a^+^annexinV^+^), leukocyte-derived (CD45^+^annexinV^+^), and monocyte-derived (CD14^+^annexinV^+^) EVs (Fig. 2c,d). EV fraction II and the EXkit fraction were used as controls in the subsequent thrombogenicity studies due to their low content or the absence of phosphatidylserine exposing EVs. The flow cytometric detection limit of 250 nm prohibited a further reliable differentiation of EVs in fraction II and in the EXkit fraction with respect to their cellular origin.

**Figure 3:**
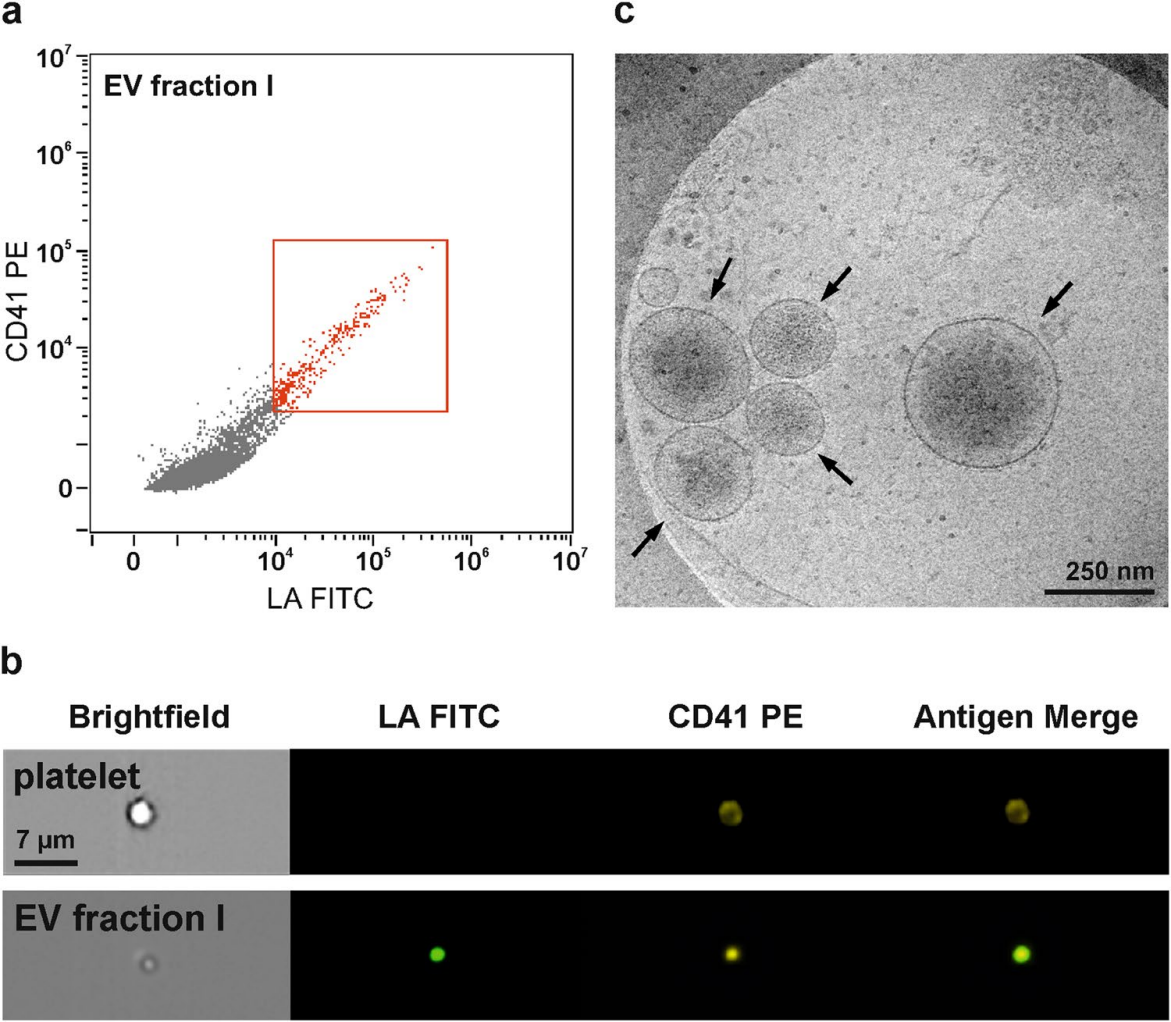
Visualization of platelet-derived EVs from EV fraction I. (**a**) EV fraction I was analyzed by imaging flow cytometry combining high speed multispectral image acquisition and automated image analysis. Phosphatidylserine exposing platelet-derived EVs were identified as lactadherin^+^ (LA^+^) and CD41^+^ events in the EV gate (red square) as described in the Methods section. (**b**) An image depicting a platelet (CD41^+^) is shown in comparison to an image of an EV from fraction I (LA^+^CD41^+^). (**c**) Cryo-electron micrographs of EV fraction I showing the presence of round, membrane-bound vesicles (black arrows) in a size range of 85–550 nm.

Mean particle sizes according to nanoparticle tracking analysis were 173 ± 15 nm for EV fraction I, 142 ± 12 nm for EV fraction II, and 100 ± 24 nm for the EXkit fraction. Imaging flow cytometry of EV fraction I confirmed the presence of CD41^+^lactadherin^+^ events in the EV size range. Cryo-electron microscopy revealed intact, spherical vesicles in a size range of 85 to 550 nm in fraction I (Fig. 3). Detergent lysis by treatment of fraction I with 0.25% TritonX-100 during staining for flow cytometry completely abolished all signals in the EV gate, confirming the presence of intact membrane vesicles (Supplementary Figure S5).

### Platelet-Derived Extracellular Vesicles Support Thrombin Generation

**Figure 4:**
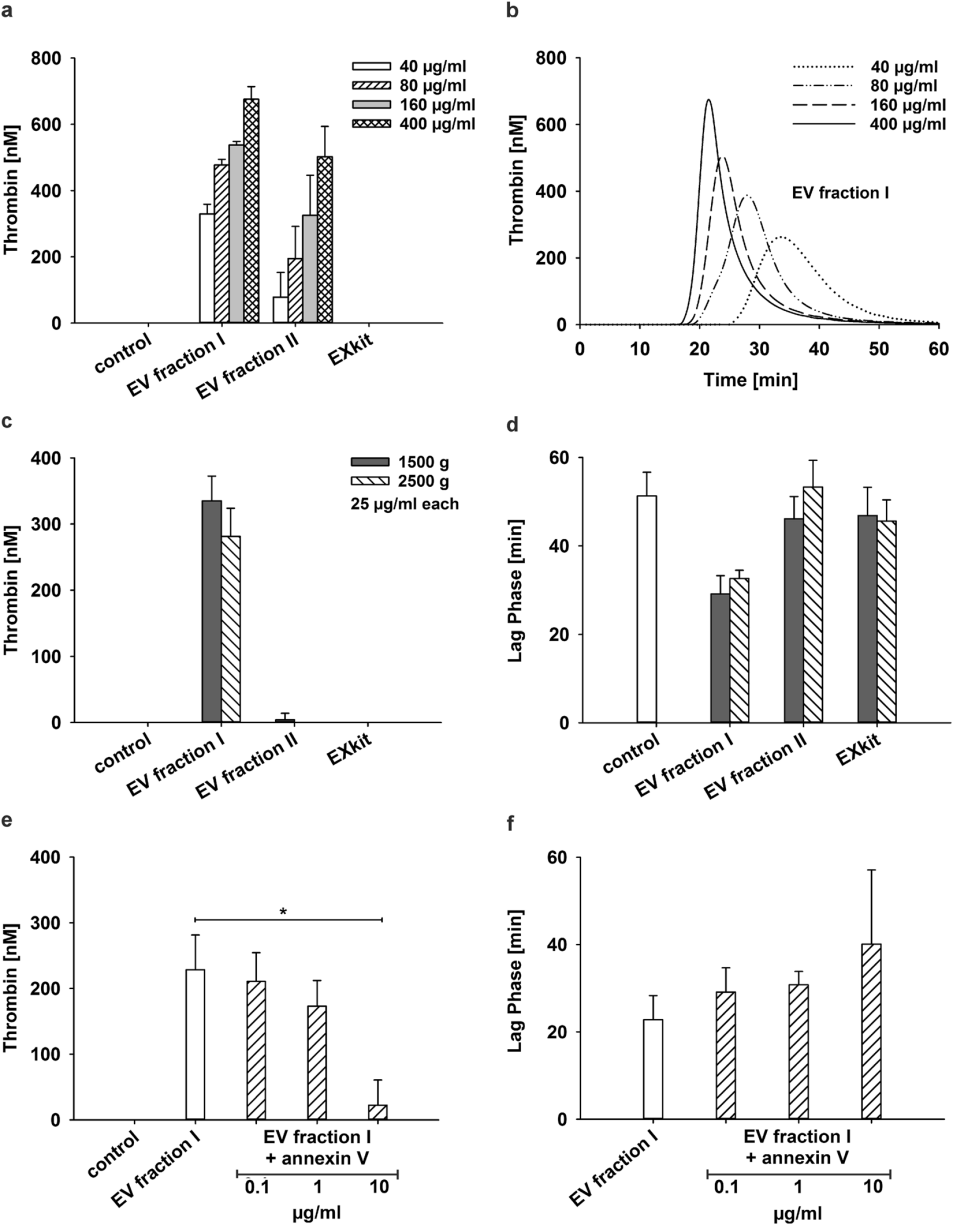
Thrombin generation by platelet-derived vesicle fractions. The thrombogenicity of isolated EVs was studied with a thrombin generation assay. Aliquots of EV suspensions (protein content as indicated in the figure) were incubated with vesicle-free plasma, and the thrombin-dependent cleavage of a fluorogenic substrate was quantified over time as described in the Methods section. (**a**) Thrombin generation after a 60 min incubation of vesicle-free plasma with increasing amounts of platelet-derived EV fractions I and II in comparison to vesicles isolated with an exosome isolation kit (EXkit). Vesicle-free plasma served as control (n = 8); (**b**) Representative curve of dose-dependent kinetics of thrombin generation induced by EV fraction I under the same conditions as shown in panel a; (**c,d**) An increase of the centrifugal force from 1,500 g to 2,500 g to remove residual platelets prior to EV isolation did not affect the amount of thrombin generation nor the lag phase to the onset of thrombin generation (n = 6); (**e,f**) Effect of phosphatidylserine blockade on thrombin generation. Pre-incubation of EV fraction I with annexin V to mask phosphatidylserine decreased thrombin generation in a dose-dependent manner (n = 6); asterisks denote P values ≤0.05.

Addition of platelet-derived EV fractions I and II to vesicle-free plasma induced thrombin generation in a dose-dependent manner (Fig. 4a,b). The time to the onset of thrombin generation (lag phase) as well as the time-to-peak decreased with increasing amounts of vesicles. EVs isolated by polymer precipitation (EXkit) did not induce thrombin generation, most likely due to their lacking phosphatidylserine exposure. To rule out a potential interference of residual platelets in the EV fractions on thrombin generation, we compared the thrombogenicity of EVs isolated after an initial centrifugation of platelet concentrates at 2,500 g instead of 1,500 g. Residual platelet counts were 2 × 10^4^ platelets/µl and 5 × 10^3^ platelets/µl for the supernatants obtained at 1,500 g and 2,500 g, respectively. In addition, the supernatant obtained at 2,500 g was filtered through 0.8 µm nylon filters to achieve complete platelet removal prior to EV isolation. As shown in Fig. 4c,d and in Supplementary Figure S1c, neither centrifugation at higher g force nor filtration prior to EV isolation had an impact on subsequent thrombin generation.

### Role of Phosphatidylserine and Tissue Factor in Thrombin Generation

**Figure 5:**
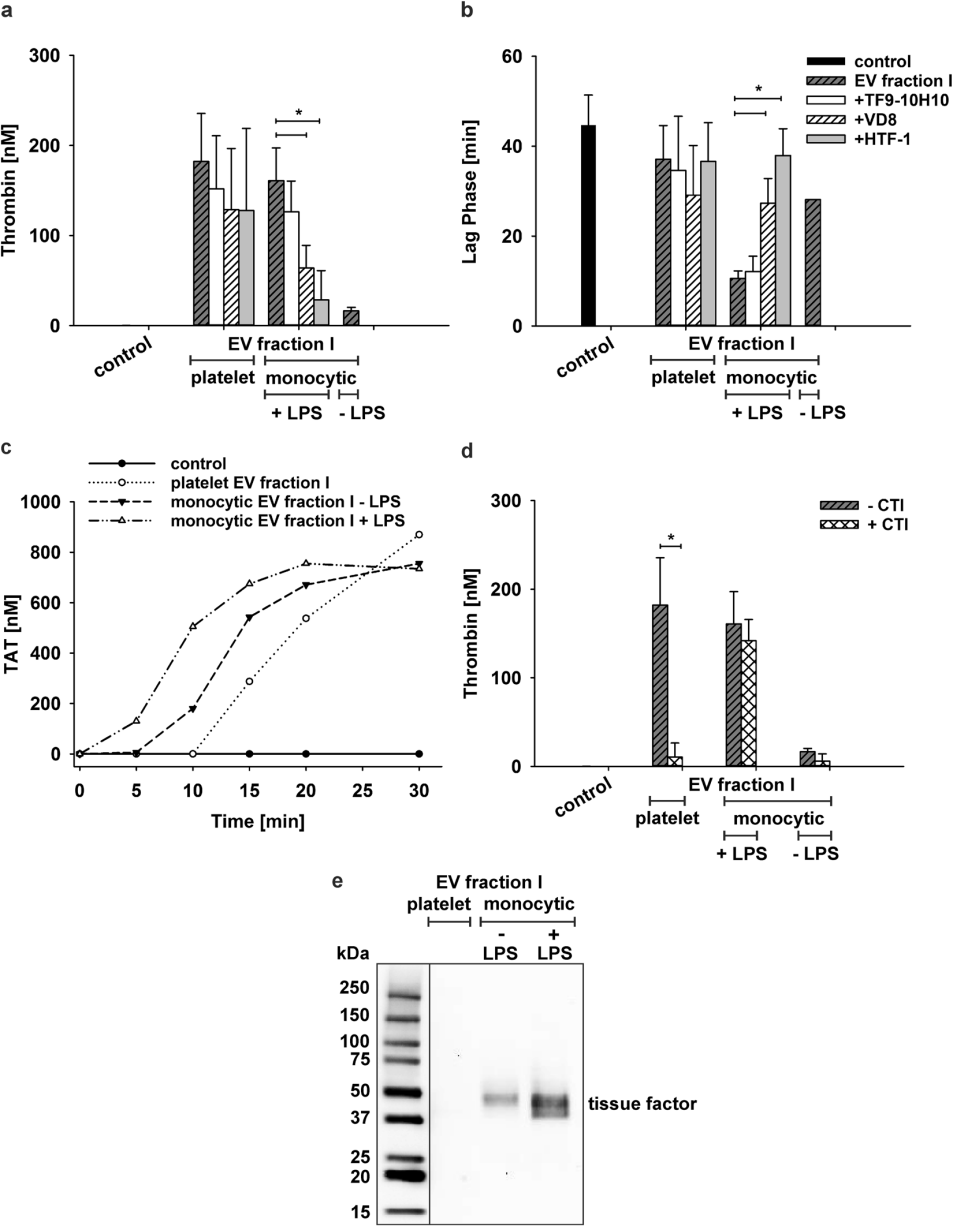
Thrombin generation induced by EVs of different cellular origin. Phosphatidylserine exposing EVs (corresponding to EV fraction I in Fig. 1) of platelet and monocytic origin were compared with respect to their thrombogenicity. (**a,b**) Thrombin generation induced by platelet-derived EVs and by EVs from unstimulated or LPS-stimulated monocytic cells (protein content 25 µg/ml each, corresponding to approximately equivalent EV concentrations). Pre-incubation of EVs from LPS-stimulated monocytic cells with the blocking anti-tissue factor antibodies HTF-1 or VD8 resulted in significantly decreased thrombin generation, while no inhibition of thrombin generation was observed with the non-blocking antibody TF9-10H10. Platelet-derived EVs induced similar amounts of thrombin as EVs from LPS-stimulated monocytic cells, albeit with a significantly longer lag phase, and pre-incubation with HTF-1 did not result in reduced thrombin generation; (**c**) Formation of thrombin-antithrombin complex (TAT) triggered by platelet-derived EVs and by EVs from LPS-stimulated or non-stimulated monocytic cells (n = 6). Vesicle-free plasma served as control. (**d**) Inhibition of the contact activation pathway by corn trypsin inhibitor (CTI) reduced thrombin generation induced by platelet-derived EVs almost to baseline levels, but had no effect on thrombin generation induced by EVs from LPS-stimulated monocytic cells (n = 9; asterisks denote P values ≤0.05. (**e**) Detection of tissue factor on Western blots of EV fraction I from platelets and from unstimulated (-LPS) and LPS-stimulated ( + LPS) monocytic cells. 10 µg of protein were loaded per lane, and the monoclonal antibody HTF-1 was used to detect tissue factor as described in the Methods section.

To assess the contribution of phosphatidylserine and tissue factor to thrombin generation, we performed blocking experiments with annexin V to mask phosphatidylserine, and with the blocking anti-tissue factor antibody HTF-1^26, 27^ to inhibit tissue factor. Pre-incubation of EV fraction I with annexin V inhibited thrombin generation in a dose-dependent manner (up to 90% using 10 µg/ml annexin V), while it did not significantly prolong the lag phase (Fig. 4e,f). Pre-treatment of platelet EV fraction I with HTF-1 at a final concentration of 10 µg/ml^27^ did not result in reduced thrombin generation, although we observed a small effect in a few donors in agreement with recent data^28^. The anti-tissue factor antibody clones VD8 (blocking) and TF9-10H10 (non-blocking) failed to inhibit thrombin generation, as well (Fig. 5a,b).

### Thrombogenicity of Platelet versus Monocytic Extracellular Vesicles

We included monocytic EVs with and without prior stimulation with lipopolysaccharide (LPS) in our experiments to assess the role of tissue factor, as the presence of tissue factor and its LPS-induced upregulation on monocytic cells are well established. EVs from LPS-stimulated monocytic cells resulted in approximately tenfold higher thrombin generation as compared to unstimulated monocytic cells (Fig. 5a) with a shorter lag phase (Fig. 5b). Western blotting showed a strong upregulation of tissue factor in EVs from LPS-stimulated as compared to unstimulated monocytic cells (Fig. 5e). Accordingly, pre-incubation of EVs from LPS-stimulated monocytic cells with anti-tissue factor antibodies HTF-1 or VD8 reduced thrombin generation by 80% and 60%, respectively, while the non-blocking antibody TF9-10H10 did not affect thrombin generation.

Remarkably, EVs from unstimulated platelets and from LPS-stimulated monocytic cells induced comparable amounts of thrombin (180 and 161 nM, respectively; Fig. 5a). The lag phase, however, was significantly longer for platelet EVs than for EVs from LPS-stimulated monocytic cells (Fig. 5b). Confirming this finding, incubation of vesicle-free plasma with EVs from LPS-stimulated monocytic cells led to significantly faster onset of thrombin-antithrombin complex (TAT) formation as compared to EVs from unstimulated monocytic cells or from platelets (Fig. 5c).

To investigate the influence of contact activation in our experimental setting, we assessed EV-induced thrombin generation in the presence of corn trypsin inhibitor (CTI), an inhibitor of FXIIa, which strongly reduced thrombin generation induced by platelet EVs (P < 0.05), but not by EVs derived from LPS-stimulated monocytic cells (Fig. 5d).

## Discussion

There is ample evidence for the role of circulating EVs in coagulation and hemostasis as well as for their involvement in various pathologies associated with thromboembolic events, such as atherosclerosis, cancer, or sepsis. The pro-coagulant activity of EVs relies on their exposure of anionic membrane phospholipids, in particular phosphatidylserine, to facilitate the assembly of coagulation complexes at the vesicle surface, while the presence of tissue factor and its potential contribution to the pro-coagulant activity of EVs remain controversial.

We chose platelet-derived EVs, which constitute the large majority of EVs in the circulation, to study the contributions of phosphatidylserine and tissue factor to their pro-coagulant activity. EVs were isolated from platelet concentrates from healthy donors by sequential centrifugation at 20,000 g (EV fraction I) and 100,000 g (EV fraction II), respectively, and the resulting vesicle fractions were characterized by flow cytometry, imaging flow cytometry, nanoparticle tracking analysis, and cryo-electron microscopy^29^. Flow cytometry confirmed the strong enrichment of platelet-derived phosphatidylserine exposing EVs, identified as CD41^+^annexinV^+^ or CD41^+^lactadherin^+^ events, in EV fraction I, while EV fraction II contained only minor amounts of CD41^+^annexinV^+^ vesicles. Samples obtained with a commercial exosome isolation kit based on polymer precipitation contained only 0.3% of phosphatidylserine exposing EVs as detected by flow cytometry and were used for comparison in thrombin generation assays.

We found that thrombin generation induced by addition of platelet-derived EVs to vesicle-free human plasma was primarily dependent on the exposure of phosphatidylserine, as supported by several lines of evidence. First, phosphatidylserine exposing EVs (fraction I) induced thrombin generation in a dose-dependent manner. EV fraction II, in which less than 10% of all EVs detected in flow cytometry exposed phosphatidylserine, induced lower amounts of thrombin than EV fraction I in assays standardized with respect to protein concentration. However, to attempt comparison based on the number of phosphatidylserine exposing vesicles, we calculated EV concentrations based on flow cytometry. With a known flow rate of 30 µl per min, an acquisition time of 3 min, and a known sample dilution factor, we could estimate the vesicle concentration for each sample and correlate it to the measured protein content. Thrombin generation assays based on comparable numbers of phosphatidylserine exposing vesicles resulted in comparable thrombin generation of EV fractions I and II (Supplementary Figure S1d). EVs isolated by polymer precipitation containing only 0.3% phosphatidylserine exposing vesicles according to flow cytometry were used for comparison and did not induce thrombin generation at all.

Second, pre-incubation of EVs with annexin V to mask phosphatidylserine inhibited thrombin generation in a dose-dependent manner, providing further support for the essential pro-coagulant effect of phosphatidylserine. Due to calcium-mediated electrostatic interactions with γ-carboxyglutamic acid containing coagulation factors, phosphatidylserine increases their local concentrations far above their plasma levels, thereby supporting their interaction at the membrane surface. Consequently, a lack of phosphatidylserine exposure results in impaired coagulation, as seen in Scott syndrome caused by defective phosphatidylserine translocation^30^.

To examine the potential contribution of tissue factor to thrombin generation by platelet EVs, we used the blocking antibody HTF-1 to inhibit tissue factor on EVs. HTF-1 exerts its blocking activity by competing with FVII for the binding site on tissue factor^26, 31^. In our experimental setting, pre-incubation of platelet-derived EVs with HTF-1 did not significantly decrease thrombin generation, which was confirmed with the blocking antibody clone VD8, indicating the absence of relevant amounts of functionally active tissue factor on platelet-derived EVs, which was in line with data obtained by Western blotting (Supplementary Figure S2a). Addition of corn trypsin inhibitor almost abolished thrombin generation induced by platelet EVs, indicating the role of contact activation to initiate coagulation in our experimental setting.

To further address the role of tissue factor, we included EVs from unstimulated and LPS-stimulated monocytic THP-1 cells in our study, as the expression of tissue factor and its LPS-induced upregulation are well established for monocytic cells. Western blotting revealed the presence of tissue factor on monocytic EVs and confirmed its strong upregulation in response to stimulation with LPS, which was associated with increased thrombin generation by EVs from LPS-stimulated *vs*. non-stimulated monocytic cells. Under non-rate-limiting conditions, platelet-derived EVs and EVs from LPS-stimulated monocytic cells induced comparable amounts of thrombin, but the lag phase to the onset of thrombin generation was considerably longer for platelet-derived EVs, as confirmed by the kinetics of TAT complex formation. In contrast to platelet-derived EVs, thrombin generation induced by LPS-stimulated monocytic EVs could be significantly reduced by their pre-incubation with the anti-tissue factor antibodies HTF-1 or VD8, confirming the presence of functionally active tissue factor on EVs from LPS-stimulated monocytic cells. Inhibition of the contact pathway by blockade of factor XII activation with corn trypsin inhibitor abolished thrombin generation in the presence of platelet-derived EVs as discussed above, but not in the presence of EVs from LPS-stimulated monocytic cells, suggesting the crucial role of tissue factor for the initiation of coagulation in the presence of LPS-stimulated monocytic cells. It remains to be clarified why platelet EVs and EVs from unstimulated monocytic cells differed with respect to their thrombogenicity in an experimental setting allowing for contact activation, i.e., in the absence of corn trypsin inhibitor. Since platelet-derived and monocytic EVs were isolated from different matrices (plasma and cell culture medium, respectively), residual plasma proteins, such as activated Factor XIIa, may be responsible for the enhanced thrombogenicity of platelet EVs as compared to monocytic EVs. Alternatively, platelet EVs and EVs from monocytic cells might differ with respect to phosphatidylserine exposure, but this assumption remains to be tested. While our current study focused on the pro-coagulant role of platelet EVs as the main source of circulating EVs and used LPS-stimulated monocytic EVs for comparison, it would be worthwhile to study the thrombogenicity of EVs from other sources, such as red blood cells, and to assess the role of different stimuli. A previous study has shown that stimulation of monocytic cells with different agonists resulted in EVs which differed at the proteomic level, but shared the same level of pro-coagulant activity^32^.

In conclusion, our data show that (1) platelet EVs support thrombin generation by the exposure of phosphatidylserine and that (2) even in the presence of phosphatidylserine exposing EVs, thrombin generation depends on an initiator. *In vivo*, tissue factor derived from stimulated monocytes in the circulation may act as initiator, e.g. in the setting of inflammation. Alternatively, activation of Factor XII may be a relevant trigger of coagulation under conditions where whole blood is in contact with biomaterial surfaces. Our findings may have further implications in the setting of transfusion medicine, as platelet concentrates contain variable amounts of EVs, dependent on the donor, the apheresis system, as well as on storage conditions and age. Platelet transfusions rich in EVs might have detrimental effects particularly in the oncological setting, since there is evidence that platelet EVs enhance the invasive potential of cancer cells^33, 34^.

## Methods

### Plasma and Platelet Concentrates

Platelet concentrates used as starting material for EV isolation were obtained from the Clinic for Blood Group Serology and Transfusion Medicine, Medical University Vienna, Austria. The study was approved by the ethics committee of the Medical University Vienna (ECS2177/2013) and conducted in accordance with the declaration of Helsinki. Written informed consent was obtained from all donors. The platelet concentrates were produced from healthy individuals eligible for single donor platelet apheresis in a blood bank setting using a Trima Accel® automated blood collection system (Version 5.0, Terumo BCT, Lakewood, CO). Platelet concentrates were stored in polyolefin bags in SSP + medium (Macopharma, Tourcoing, France) containing 69.3 mM sodium chloride, 10.8 mM citrate, 32.5 mM acetate, 28.2 mM phosphate, 5.0 mM potassium, and 1.5 mM magnesium at a ratio of 80% SSP + and 20% plasma, and were used for EV isolation within 1–3 days. Each batch was characterized by blood cell counting (Sysmex KX-21 N, Sysmex, Neumuenster, Germany) and by flow cytometric analysis of platelet activation (CD62P surface expression). In addition, total EV counts per µl and the distribution of EVs with respect to their platelet, erythrocyte, and leukocyte origin were determined by flow cytometry as described below.

For thrombin generation assays, whole blood was freshly drawn from healthy adult volunteers. Plasma was generated from whole blood by centrifugation at 2,000 g for 10 min at room temperature and depleted of remaining platelets and vesicles by centrifugation (100,000 g, 1 h, 4 °C) followed by filtration (Millex VV 0.1 µm filter unit, Merck Millipore, Darmstadt, Germany). For cell culture experiments, human AB serum was purchased from Sigma Aldrich (St. Louis, MO) or from Octapharma (Vienna, Austria), centrifuged (20,000 g, 30 min, 4 °C), and sterile filtered as described above.

### Cells and Cell Culture

The human monocytic cell line THP-1 was obtained from the American Type Culture Collection (ATCC) and maintained as described^35^. Cell culture medium RPMI-1640, 4-(2-hydroxyethyl)-1-piperazineethanesulfonic acid (HEPES), penicillin-streptomycin, fetal bovine serum (FBS), and lipopolysaccharide (LPS) from *E. coli* (055:B5) were from Sigma Aldrich.

### Chemicals and Reagents

Phosphate Buffered Saline (PBS) without Ca^2+^ and Mg^2+^ was purchased from Biochrom (Berlin, Germany). Gels (4–20%, Mini-PROTEAN), molecular weight markers (Precision plus Western C protein standard), and reagents for sodium dodecyl sulfate polyacrylamide gel electrophoresis (SDS-PAGE), membranes (Trans-Blot transfer pack, nitrocellulose), as well as reagents for Western Blotting and the DC Protein Assay kit were obtained from Bio-Rad (Hercules, CA, USA). Annexin V binding buffer was from Becton Dickinson (Franklin Lakes, NJ).

### Tissue Factor Antibodies

The anti-tissue factor mouse monoclonal antibody (mAb) HTF-1 (eBioscience, San Diego, CA) was used to inhibit tissue factor-dependent thrombin generation^26^. The non-blocking anti-tissue factor mouse mAb TF9-10H10 (Merck) was employed as control in blocking experiments^36^. The mouse mAb VD8 was from Sekisui Diagnostics GmbH (Pfungstadt, Germany)^24^.

### Isolation of Extracellular Vesicles

EVs were isolated from fresh platelet concentrates (90 ml) as shown in Fig. 1. After removal of platelets by centrifugation (1,500 g or 2,500 g, 15 min, RT), EVs were pelleted at 20,000 g (30 min, 4 °C) using a Sorvall Evolution RC ultracentrifuge, Rotor SS-34 (Thermo Fisher Scientific, Waltham, MA). The resulting pellet (EV fraction I) was washed with sterile PBS, re-centrifuged at 20,000 g (30 min, 4 °C), re-suspended in PBS to a final protein concentration of 4 mg/ml, aliquoted, and stored at −80 °C until further use. The supernatant was centrifuged at 100,000 g (60 min, 4 °C), using an Optima MAX ultracentrifuge, MLA-80 Rotor (Beckman Coulter, Brea, CA). The resulting pellet (EV fraction II) was washed in PBS and re-centrifuged, suspended at a protein concentration of 4 mg/ml, aliquoted, and stored at −80 °C until further use. Alternatively, EVs were precipitated directly from the supernatant of the platelet concentrate using the Total Exosome Isolation Kit from plasma (Invitrogen, Carlsbad, CA) according to the instructions of the manufacturer to yield the EXkit fraction.

### Stimulation of Monocytic THP-1 Cells

THP-1 cells (1 × 10^6^ cells/ml) were stimulated with 5 µg/ml LPS for 4 h at 37 °C in the presence of 10% of human AB serum or left untreated. After stimulation, cells were removed by centrifugation (500 g, 5 min, 4 °C), the supernatant was collected and re-centrifuged at 1,500 g for 10 min to remove cellular debris, and the resulting supernatants were used to isolate EV fraction I as described for platelet EVs above.

### Flow Cytometric Characterization of Platelet Concentrates and EV Fractions

Platelet concentrates and EV fractions were characterized using a Gallios Flow Cytometer (Beckman Coulter). Calibration was performed with fluorescent beads (0.1, 0.3, 0.5, and 0.9 µm; Megamix Plus FSC, Biocytex, Marseille, France) according to the instructions of the manufacturer, and the EV gate was set above the 0.9 µm bead cloud as previously described^4, 37, 38^ and as shown in Fig. 2. Samples were diluted 1:500 in sterile filtered annexin V binding buffer prior to analysis. Staining of platelet-derived vesicles was performed with fluorescein isothiocyanate (FITC)-conjugated annexin V as marker for phosphatidylserine in combination with a phycocyanin (PC7)-conjugated anti-CD41 mAb as platelet marker (all from Beckman Coulter). To confirm that the signals in the EV fraction were indeed dependent on the presence of intact EVs, a detergent lysis control was performed by treatment of the EV fraction with 0.25% TritonX-100 during staining (Supplementary Figure S5).

To evaluate the presence of EVs derived from other blood cells, we used phycoerythrin (PE)-conjugated anti-CD14 as monocyte marker, APC-conjugated anti-CD235a as erythrocyte marker, and pacific blue (PB)-conjugated anti-CD45 as leukocyte marker.

Platelet activation was analyzed using PE-conjugated anti-CD41 as platelet marker and FITC-conjugated anti-CD62P as activation marker. Monocytic EVs were stained with PE-conjugated annexin V in combination with PB-conjugated anti-CD45 as leukocyte marker (all from Beckman Coulter). Buffer controls, isotype controls, and single stainings of specific monoclonal antibodies are shown in Supplementary Figure S4b. Samples were measured for 3 min at a flow rate of 30 µl per min and data were analyzed using the Kaluza Software (Beckman Coulter).

### Visualization of EVs by Imaging Flow Cytometry

EV fractions were analyzed with an ImageStream^x^ MkII cytometer (INSPIRE v6.2, Amnis, Seattle, WA) with low flow rate (high sensitivity) using 60x magnification, providing a pixel size of 0.3 µm x 0.3 µm. Samples were diluted 1:100 in sterile filtered PBS. Staining of platelet-derived vesicles was performed with FITC-conjugated lactadherin (Haematologic Technologies Inc., Essex Junction, VT) as marker for phosphatidylserine exposing EVs in combination with PE-conjugated anti-CD41 as platelet marker (Beckman Coulter). Events were first selected on focused events (Gradient RMS_BF > 0.5). Subsequently, singlets were gated versus doublets in a new plot using Area_BF (Ch01) versus Aspect Ratio_BF (Ch01). Singlets were defined visually and correlated with an aspect ratio >0.8 and an area of 10–35. Singlets were further plotted to lactadherin FITC (Intensity MC_Ch02) versus CD41 PE (Intensity MC_Ch03) and EVs appeared as small, spherical FITC and PE double positive events.

### Cryo-Electron Microscopy

Quantifoil (Quantifoil Micro Tools GmbH, Großlöbichau, Germany) holey carbon copper grids (Cu 400 mesh, R 1.2/1.3; hole diameter: 1.2 µm, hole spacing: 1.3 µm) were glow discharged for 60 sec and loaded into a Leica EM GP grid plunger (Leica Microsystems, Wetzlar, Germany). Forceps with grids were mounted into the plunger and loaded with 4 µl of EV fraction I (400 µg/ml final concentration). After a pre-incubation of 30 sec at 20 °C and 70% humidity, samples were blotted for 2 or 4 sec and rapidly frozen in ethane at approximately −180 °C for vitrification. Grids were stored under liquid nitrogen. Cryo samples were examined using a Tecnai F30 Polara transmission electron microscope (FEI, Hillsboro, OR) with a 300 kV field emission gun. The microscope was operated under low dose conditions using SerialEM, and image acquisition utilized a defocus of 8 µm and 20 e/A2 electron dose. Digital images were recorded with a Orius 2k x 2k CCD camera (FEI) at 9400 fold magnification and a resulting pixel size of 0.62 nm.

### Nanoparticle Tracking Analysis

The size distribution of EV fractions was analyzed by nanoparticle tracking analysis (Nanosight NS-300, Malvern Instruments, Malvern, UK). Samples were diluted in PBS to a final protein concentration of 0.8–1 µg/ml and injected manually. Data were acquired at room temperature, camera level 15, frame rate 24, and analyzed using the NTA 3.0 software.

### Thrombin Generation

The thrombogenicity of EV fractions was assessed with a thrombin generation assay (Technoclone, Vienna, Austria) based on the thrombin-dependent cleavage of a fluorogenic substrate over time. Aliquots (10 µl) of EV suspensions (40, 80, 160, 400 µg protein/ml) were incubated with 40 µl of vesicle-free human plasma, and the kinetic reading was initiated upon addition of 50 µl of fluorogenic substrate solution containing 15 mM CaCl_2_. Measurements were recorded at 37 °C for 1 h with 1 min intervals using the Synergy 2 reader (Bio-Tek Instruments Inc., Winooski, VT) at 360/460 nm. Data were analyzed with the Bio-Tek Gen5 software. Vesicle-free plasma served as negative control. To assess the role of phosphatidylserine in thrombin generation, EV fraction I (36 µl; protein content 25 µg/ml) was pre-incubated for 30 min at RT with 4 µl of annexin V (10, 1, and 0.1 µg/ml final concentration, dilutions made in annexin V binding buffer) from human placenta (Sigma Aldrich). To study tissue factor-dependent thrombin generation, EV fractions I from platelets and monocytic cells, respectively (protein content 25 µg/ml) were pre-incubated with the blocking anti-tissue factor antibody clones HTF-1 or VD8, or with the non-blocking anti-tissue factor antibody TF9-10H10 as control, all at a final concentration of 10 µg/ml. Thrombin generation was additionally studied in plasma anticoagulated with 11 mM sodium citrate and 50 µg/ml corn trypsin inhibitor (Haematologic Technologies) to exclude contact activation.

### Formation of Thrombin-Antithrombin Complex

Complementing the thrombin generation assay, the thrombogenicity of EVs was studied by TAT formation. Human plasma was incubated with EV fraction I (25 µg protein/ml) from monocytic THP-1 cells or from platelets in the presence of 7 mM CaCl_2_ at 37 °C for 0, 15, 30, and 60 min. The reaction was terminated by addition of 10 mM EDTA, and the TAT complex was quantified using a sandwich immunoassay (Enzygnost TAT micro, DADE Behring, Marburg, Germany). Vesicle-free plasma incubated under identical conditions served as negative control.

### Detection of Tissue Factor by Western Blotting

The presence of tissue factor in platelet-derived EV fractions as well as in EVs from LPS-stimulated and unstimulated monocytic cells was assessed by Western blotting. Samples (10 µg total protein per lane) were separated in 4–20% gels under reducing conditions and blotted onto nitrocellulose membranes. Membranes were incubated with the anti-tissue factor mouse monoclonal HTF-1 at a final concentration of 1 µg/ml and developed with the Western Breeze chemiluminescent kit (Invitrogen).

### Statistical Analysis

For statistical analysis, the IBM SPSS Statistics software (version 20, IBM Corporation, NY) was used. The Mann-Whitney-U test was used to compare two data groups. P values ≤0.05 were considered as statistically significant.

### Data availability

The datasets generated during and/or analyzed during the current study are available from the corresponding author on reasonable request.

## Electronic supplementary material

Supplementary Figures
