# Supplementary material for: Different Potential of Extracellular Vesicles to Support Thrombin Generation: Contributions of Phosphatidylserine, Tissue Factor, and Cellular Origin
Source: Sci Rep. 2017 Jul 26;7:6522. doi: 10.1038/s41598-017-03262-2 (PMC5529579; doi:10.1038/s41598-017-03262-2)
Supplement: Supplementary file 1 — Supplementary Figures [file 41598_2017_3262_MOESM1_ESM.pdf]

Different Potential of Extracellular Vesicles to Support Thrombin Generation: Contributions of Phosphatidylserine, Tissue Factor, and Cellular Origin

Carla Tripisciano[1], René Weiss[1], Tanja Eichhorn[1], Andreas Spittler[2], Thomas Heuser[3], Michael Bernhard Fischer[1,4#], Viktoria Weber[1,4#]

[1]Christian Doppler Laboratory for Innovative Therapy Approaches in Sepsis, Danube University Krems, Dr.-Karl-Dorrek-Strasse 30, 3500 Krems, Austria
[2]Core Facility Flow Cytometry & Surgical Research Laboratories, Medical University of Vienna, Lazarettgasse 14, 1090 Vienna, Austria
[3]Electron Microscopy Facility, Vienna Biocenter Core Facilities, Dr.-Bohr-Gasse 3, 1030 Vienna, Austria
[4]Center for Biomedical Technology, Department for Health Sciences and Biomedicine, Danube University Krems, Dr.-Karl-Dorrek-Strasse 30, 3500 Krems, Austria

[#] equally contributing last authors

To whom correspondence should be addressed: Prof. Viktoria Weber, Danube University Krems, Dr.-Karl-Dorrek-Strasse 30, 3500 Krems, Austria, Tel.: +43 2732 893 2632; fax: +43 2732 893 4600; e-mail: viktoria.weber@donau-uni.ac.at

Supplementary Figure S1

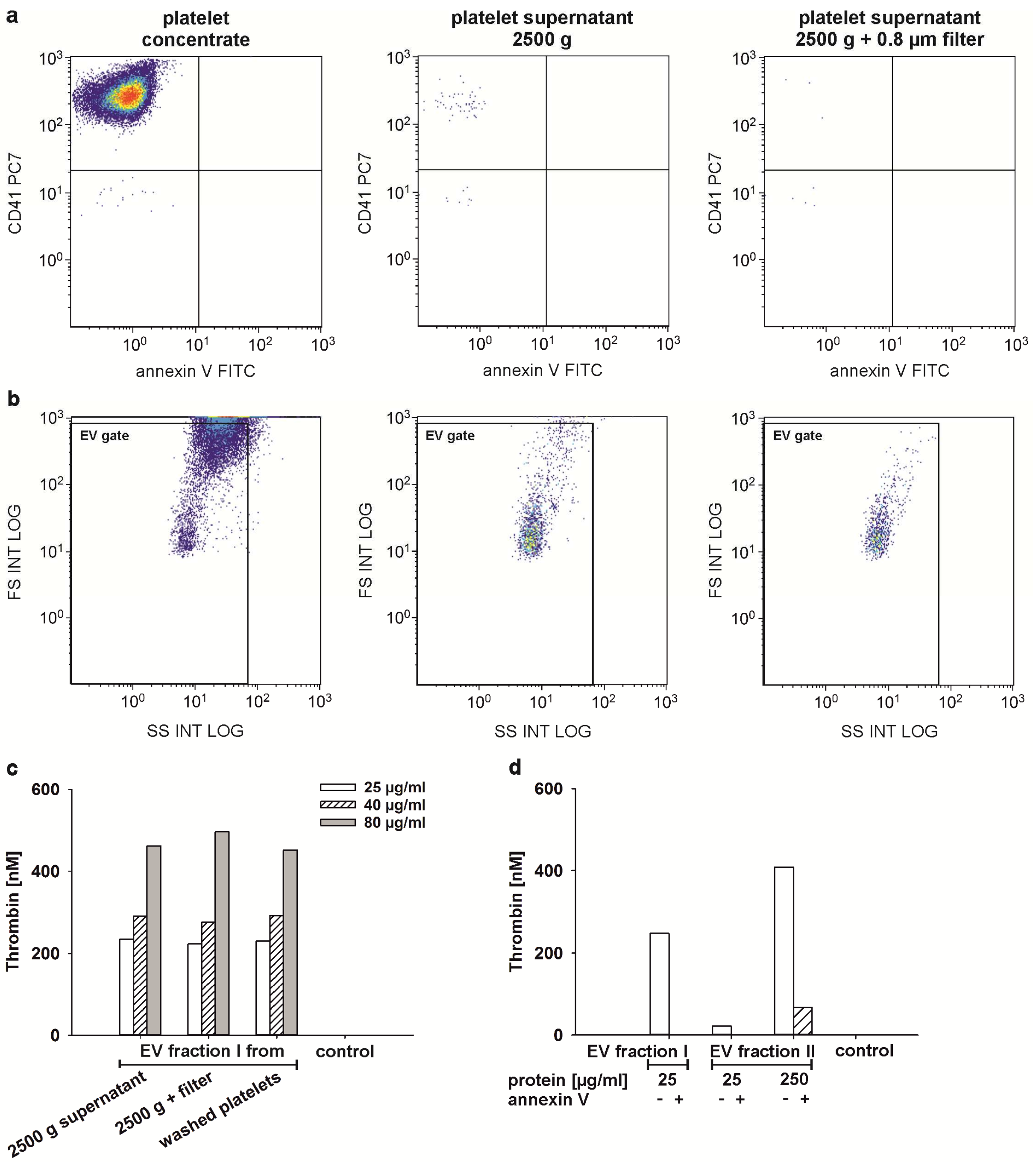

Supplementary Figure S1: Characterization of platelet concentrate and platelet supernatants using flow cytometry, and thrombin generation assays with extracellular vesicles (EVs) obtained under different experimental conditions.

In addition to the standard protocol for EV isolation (main manuscript, Figure 1), platelet supernatants were obtained by centrifugation of platelet concentrate at higher g force (2,500 g), and residual platelets were depleted by filtration (0.8 µm nylon filters, Merck Millipore, Tullagreen, Ireland). **(a)** The platelet concentrate and the supernatants were characterized by flow cytometry using CD41 as platelet marker and annexin V as marker for phosphatidylserine, showing the depletion of platelets

from the 2,500 g supernatant and from the filtered supernatant. **(b)** The EV gate was set as described in the Methods section and forward scatter *vs.* side scatter (FS *vs.* SS) dot plots for platelet concentrate (left), platelet supernatant obtained by centrifugation of platelet concentrate at 2,500 g (middle), and platelet supernatant obtained by centrifugation at 2,500 g plus filtration (right) are shown. **(c)** Thrombin generation induced by EVs isolated from the 2,500 g supernatant with and without filtration. EVs isolated from the 2,500 g supernatant or from the filtered 2,500 g supernatant did not differ from EVs isolated from the 1,500 g supernatant (standard isolation protocol, Figure 1, main manuscript) regarding thrombin generation, excluding a potential influence of residual platelets on thrombin generation. To further exclude any influence of plasma-derived contaminants, fresh platelet concentrates were centrifuged at 2,500 g, and platelets were resuspended in storage medium SSP+ to obtain washed platelets, which were stored for 2 days prior to isolation of EVs. EVs from these washed platelets did not differ regarding thrombin generation from EVs isolated with the standard protocol. **(d)** Dose-dependent thrombin generation by platelet EV fractions I and II. In general, thrombin generation assays were standardized to equal amounts of protein in the vesicle fractions. To attempt comparison based on vesicle numbers, we calculated EV concentrations based on flow cytometry (acquisition time of 3 min, flow rate of 30 µl per min, known sample dilution factor) and correlated the approximate EV numbers to protein content for each sample. According to this approximation, platelet EV fraction I contained 4×10^5 EVs per µg of protein, while EV fraction II contained 2.3×10^4 EVs per µg of protein. We assessed thrombin generation using equal amounts of protein from EV fractions I and II (25 µg each) as well as a 10-fold excess of EV fraction II (250 µg of protein) to yield samples containing approximately equal concentrations of phosphatidylserine exposing EVs, resulting in comparable thrombin generation of the two samples, which could be completely blocked using annexin V (10 µg/ml). Vesicle-free plasma was used as control.

Supplementary Figure S2

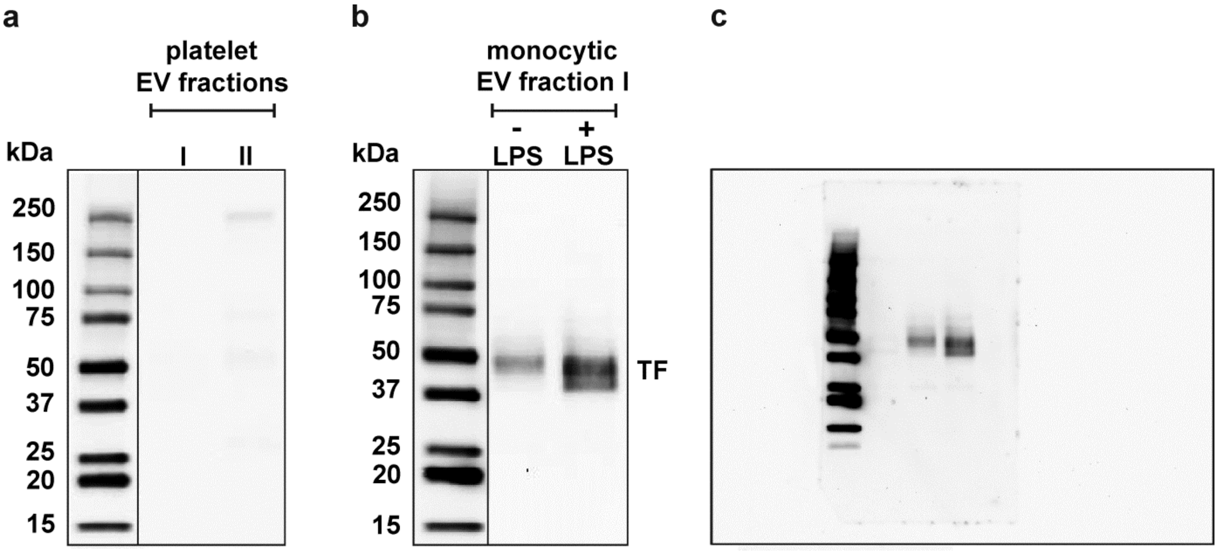

Supplementary Figure S2: Detection of tissue factor (TF) on Western blots of **(a)** platelet and **(b)** monocytic EV fractions, showing the absence of TF from platelet EV fractions I and II, and the LPS-induced upregulation of TF on monocytic EVs. 10 µg of protein were loaded per lane, and blots were probed with the anti-TF antibody HTF-1 as described in the Methods section of the main manuscript. **(c)** Uncropped original scan of the blot depicted in Figure 5e of the main manuscript.

Supplementary Figures S3

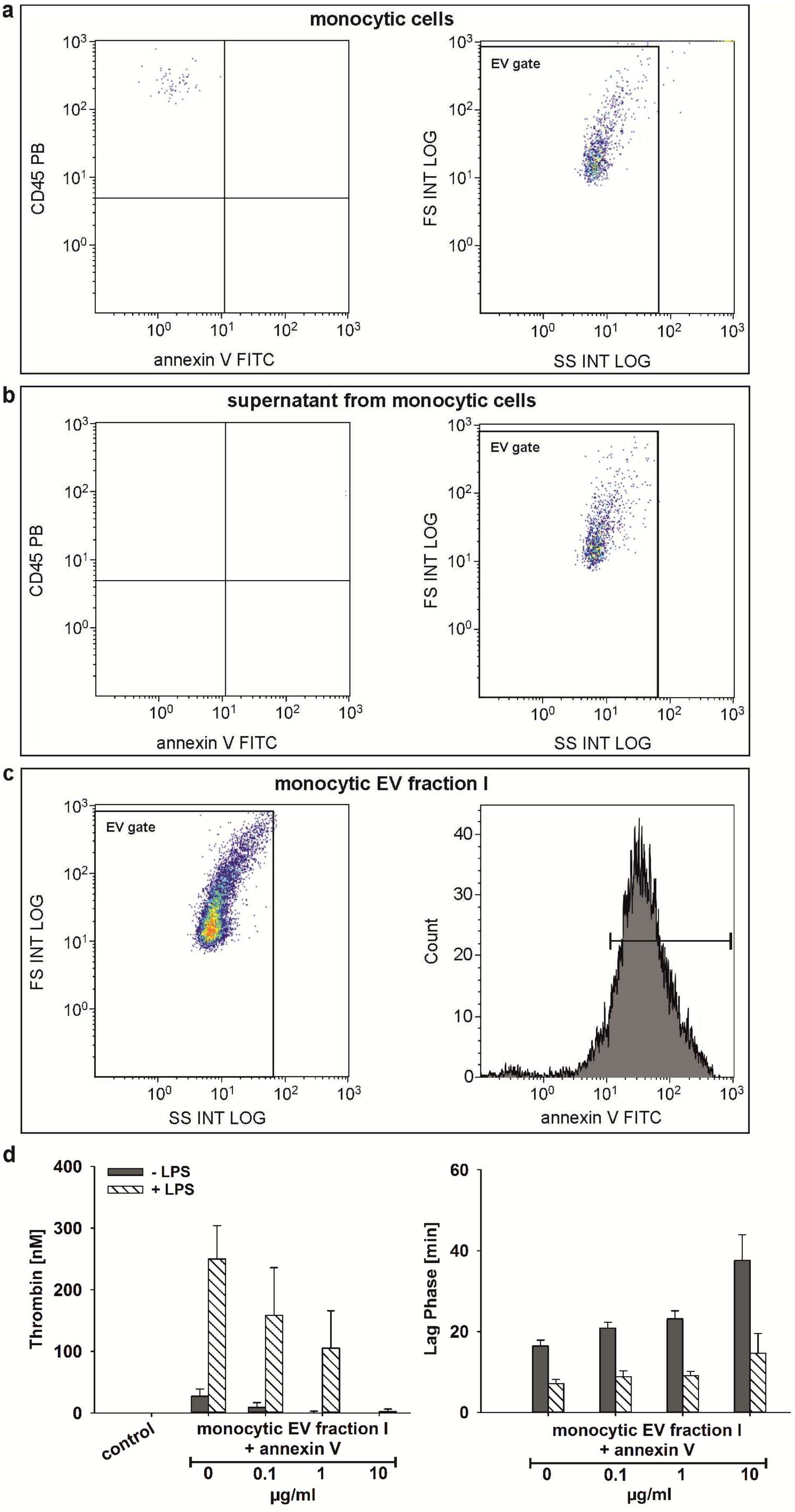

Supplementary Figure S3: Flow cytometric characterization of monocytic cells, monocyte supernatants, and monocytic EV fraction I. **(a)** Monocytic THP-1 cells were characterized using CD45 as leukocyte marker and annexin V as marker for phosphatidylserine (left panel). The EV gate was set as described in the Methods section of the main manuscript, as shown in the FS *vs.* SS plot (right panel). **(b)** Monocytic cells were pelleted by centrifugation at 1,500 g. The depletion of monocytic cells from the supernatants (left panel) and the presence of phosphatidylserine exposing EVs (right panel) were confirmed by flow cytometry. **(c)** EVs obtained by centrifugation of the monocyte supernatant at 20,000 g are shown by FS *vs.* SS plot (left panel), and exposure of phosphatidylserine was confirmed with annexin V (right panel). **(d)** EVs from LPS-stimulated monocytic cells induced thrombin generation (compare main manuscript, Figure 5a). Thrombin generation could be dose-dependently inhibited by annexin V, as shown in panel d above. EVs from unstimulated monocytic cells induced only minimal thrombin generation and are shown for comparison (n=3). A protein concentration of 25µg/ml was used in all experiments.

Supplementary Figure S4

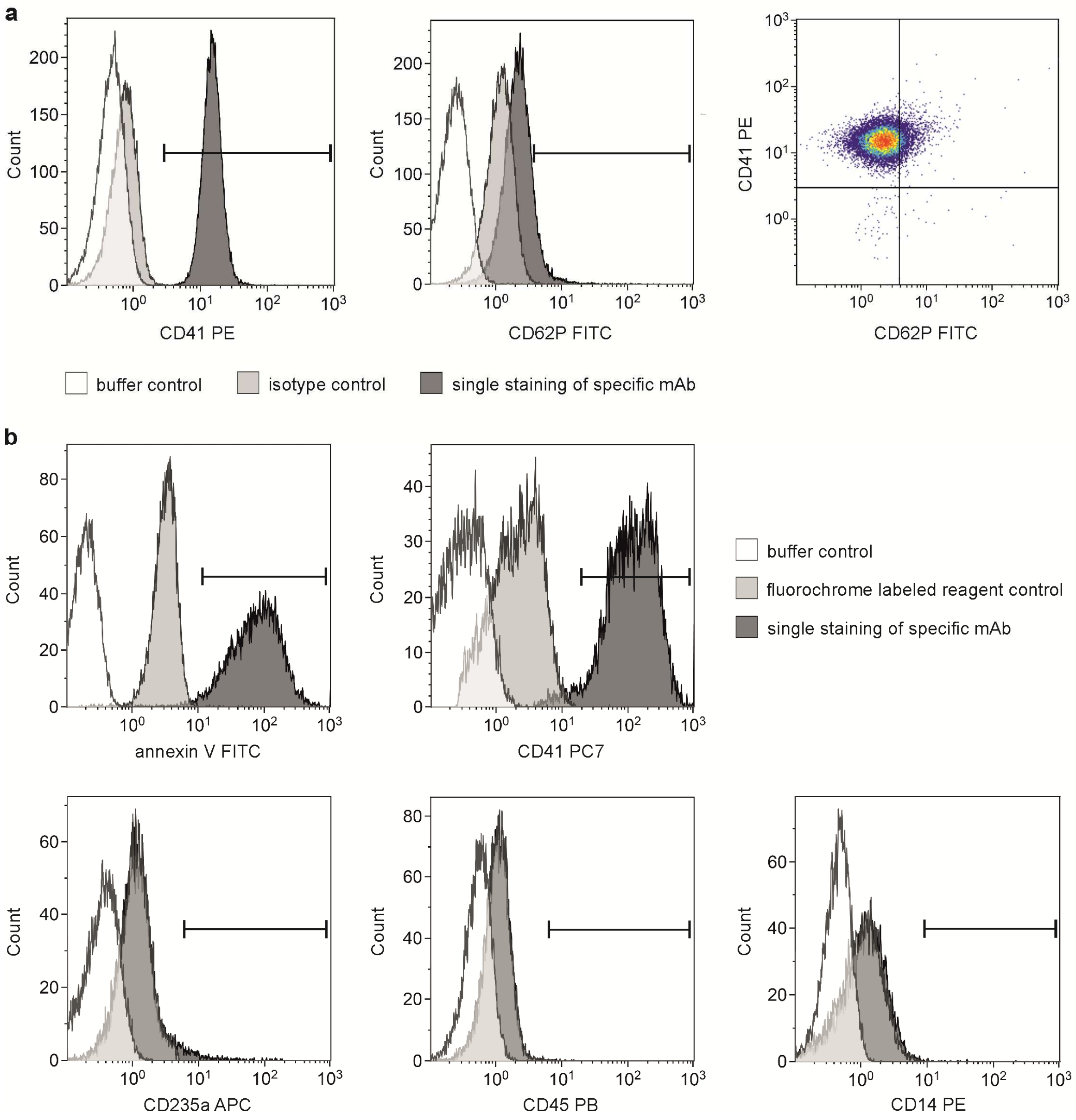

Supplementary Figure S4: Control experiments for flow cytometric characterization of platelet concentrates and extracellular vesicle fractions. **(a)** Platelet activation was monitored by flow cytometric analysis of phycoerythrin (PE)-conjugated anti-CD41 as platelet marker and fluorescein isothiocyanate (FITC)-conjugated anti-CD62P as a platelet activation marker. The respective buffer controls, isotype controls, and single stainings are shown. **(b)** As described in the Methods section, staining of platelet-derived EVs was performed with (FITC)-conjugated annexin V as marker for phosphatidylserine in combination with a phycocyanin (PC7)-conjugated anti-CD41 monoclonal antibody (mAb) as platelet marker. To specify the presence of EVs from other blood cells, phycoerythrin (PE)-conjugated anti-CD14 was used as monocyte marker, APC-conjugated anti-CD235a as erythrocyte marker, and pacific blue (PB)-conjugated anti-CD45 as leukocyte marker. The respective buffer controls, fluorochrome labeled reagent controls, and single stainings are shown. Bars indicate positive expression.

Supplementary Figure S5

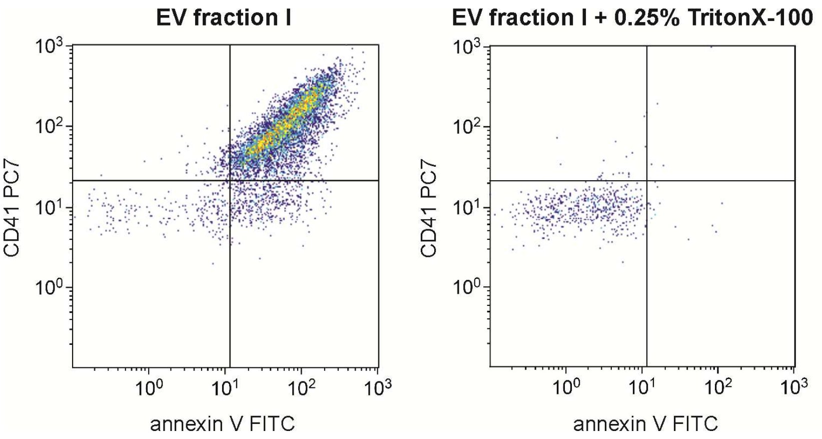

Supplementary Figure S5: Platelet EV fraction I (see Figure 1, main manuscript) was treated with 0.25% TritonX-100 during staining as detergent lysis control, abolishing all signals in the EV gate and confirming the presence of intact vesicles.
